# Complete genome sequence of *Kozakia baliensis* LTH 7215, a newly isolated strain from grape must

**DOI:** 10.1128/mra.00784-24

**Published:** 2024-10-21

**Authors:** Dor Zipori, Merlin Brychcy, Herbert Schmidt

**Affiliations:** 1 Department of Food Microbiology and Hygiene, Institute of Food Science and Microbiology, University of Hohenheim, Stuttgart, Germany; Indiana University, Bloomington, Bloomington, Indiana, USA

**Keywords:** *Kozakia baliensis*, exopolysaccharide, genome

## Abstract

We report the complete genome sequence of the novel *Kozakia baliensis* strain LTH 7215, isolated from grape must. This strain exhibits a mucoid phenotype on solid medium, associated with exopolysaccharide production. The high-quality and full-length genome sequence was obtained using PacBio Sequencing technology, refined with Illumina sequencing.

## ANNOUNCEMENT

*Kozakia baliensis* was classified as a new species belonging to the Acetobacteraceae in 2002 (1). The bacterium is known to produce exopolysaccharides (EPS), which may have industrial applications (2, 3). *K. baliensis* LTH 7215 was isolated from grape must, produced at the University of Hohenheim. The strain exhibits an EPS-associated mucoid phenotype on solid media.

To isolate the bacterial strain, 10 g grape must be mixed with 100 mL of sterile 0.9% (wt/vol) NaCl solution. The resulting suspension was plated onto MRS agar (Merck KGaA, Germany) and incubated for 72 h at 30°C. A single mucoid colony was selected for further analysis. Genetic identification of the isolated strain was carried out through the 16S rRNA gene following the method described by Sterr et al. (4), with some modifications. Genomic DNA was extracted using the DNeasy Blood&Tissue Kit (Qiagen, Germany). Fast Alkaline Phosphatase (Thermo Fisher Scientific, USA) was used to purify the PCR products. Sanger sequencing of the purified PCR products was performed by Eurofins (Germany), utilizing the same primer pair used for the PCR amplification.

*K. baliensis* LTH 7215 was cultured on Yeast Extract, Peptone, and Mannitol (YPM) agar plates, containing (in g/L): five yeast extract, three meat peptone, 25 D-mannitol, and 12 agar. For liquid culture, one bacterial colony was inoculated into 10 mL of YPM broth and incubated at 30°C while shaking overnight at 180 rpm.

DNA extraction and genome sequencing were performed by Biomarker Technologies (BMK) GmbH (Münster, Germany). Whole-genome sequencing of *the* isolated strain was performed using a hybrid approach that combined Pacific Biosciences (PacBio) Revio and Illumina NovaSeq X Plus technologies.

DNA was extracted from the bacterial pellet using the Guide S96 Magnetic Universal DNA Kit (Tiangen, China). For Illumina sequencing, a library was constructed through enzymatic fragmentation and PCR amplification using VAHTS Universal Plus DNA Library Prep Kit for Illumina (Vazyme, China). PCR products were purified using VAHTSTM DNA Clean Beads (Vazyme, China). The library was sequenced on the Illumina NovaSeq X Plus platform using a paired-end 150 strategy. Quality control was done using fastp (5) with “-q 10u 50 -y -g -Y 10 -e 20L 100 -b 150 -B 150” settings, fastuniq (6) and Trimmomatic (7) with parameters “LEADING:3 TRAILING:3 SLIDINGWINDOW:50:20 MINLEN:10.”

For PacBio sequencing, DNA was fragmented using a g-TUBE (Covaris, USA) and nick- and end-repaired after. SMRTbell adapters were then ligated to the prepared DNA fragments. The ligation products were treated with exonuclease. Fragments of the desired size were selected using BluePippin technology. The Revio Polymerase Kit (PacBio, USA) was utilized to bind the primer and polymerase to the library and the reaction was purified using SMRTbell cleanup beads (PacBio, USA). Sequencing using a PacBio Revio (PacBio, USA). Reads were quality controlled using ccs (PacBio) with parameters “--min-passes 3--min-rq 0.99 --minLength 500,” subsequently reads under 2,000 bp were removed. PacBio data were assembled using Hifiasm (v0.12) (8), and the assembly was polished with Illumina reads using Pilon (v1.22) (9). The genome sequence was annotated using the PGAP (v6.6) (10). Circularization was confirmed using Circlator 1.5.5 (11). Essential quality metrics and genome properties are shown in Table 1.

**TABLE 1:** Quality metrics and genome properties of *Kozakia baliensis* LTH 7215

Genome length (bp)	3,350,345
Chromosome length (bp)	2,893,325
Plasmid lengths (bp)	208,094; 124,118; 65,585; 53,127; 6,096
GC content (%)	57.26
Total reads Illumina	4,047,754
Total reads PacBio	237,861
N50 PacBio	10,221
Coverage	330.87
Number of coding genes	3,216
Number of coding sequences	3,028

Using the CAZy database for carbohydrate-active enzymes (12), we identified 68 genes encoding glycosyltransferases, a class of enzymes involved in EPS production (13–15). Further genome analysis and *in vitro* studies will enable a more detailed characterization of the strain and the EPS it produces.

## Data Availability

This complete genome sequence has been deposited in GenBank data base under the accession numbers CP158988-CP158993. The associated BioProject, SRA, and BioSample accession numbers are PRJNA1126072, SRR29606854-SRR29606855, and SAMN41920488, respectively.
